# A Treatise on Diseases and Injuries of the Nerves

**Published:** 1834-04-01

**Authors:** 


A Treatise on Diseases and Injuries of the Nerves. By *Joseph Swan.* A new Edition, very considerably enlarged. 8vo. pp. 351. Ten Copper-plates. London, 1834.

The motto of Mr. Swan is expressive of his modesty and an earnest of his merit. The able investigator of the minute anatomy of the nervous system has approached its diseases and its injuries with a deprecatory quotation from Cicero's Epistles :—

" Non scribo hoc temere. Quo minus familiaris sum, hoc sum ad investigandum curiosior."

Mr. Swan observes that the first edition of the present work had been long disposed of—that many communications had been made to the profession in the last two years on the subject of diseases of the nerves—that these, though numerous and not devoid of value, were insufficient to fill the gaps that intersect this field of science—and finally that *his* aim was partly to supply those obvious deficiencies, principally to direct and to stimulate enquiry. On the whole, this volume might fairly claim to be viewed in the light of a new book, rather than in that of a new edition.

It consists of sixteen chapters, some of which assume a physiological, the majority a pathological aspect. The arrangement of the author is not altogether free from objection, and we take the liberty of deviating from it in the following notice of the contents of the work. This is merely a matter of convenience, and whilst our readers will probably gain, Mr. Swan can scarcely lose.

The chapter at which we shall commence is Mr. Swan's eleventh. It is headed, " An experimental inquiry into the process Nature employs for repairing wounds of nerves." The details of twenty-five experiments are given, and the reader is presented with an account of the effects occasioned by division of the sciatic and the pneumo-gastric nerves, by excision of a portion of those trunks, and by ligatures applied to them. For the actual experiments we refer the reader to the work itself, and merely cast a hurried glance at their general results. Mr. Swan observes :—

" It will be seen from these experiments, in the first place, that after a division of a nerve, the extremities of the divided portions become enlarged and more vascular, but especially the upper portion; and coagulable lymph, having the appearance of white of egg, is effused, which soon becomes vascular. In a few days the coagulable lymph from each portion becomes united, and anastomoses form between the blood vessels; the coagulable lymph gradually assumes a firmer texture, and the number of the blood vessels diminishes, and the newly-formed substance appears to contract, like all other cicatrices, so as to bring the extremities of the divided portions nearer and nearer to each other. It is difficult to determine from an experiment on the limb of an animal the exact time at which the nerve again performs its functions. In eight weeks after the division of the sciatic nerve, I have observed a rabbit to be in some degree improved in the use of its leg, but at the end of eighteen weeks it was not perfect. When the nerves of the leg of a horse have been divided just above the foot, they are sufficiently restored to perform their functions in a very great degree in six or eight weeks; but it must be observed that these nerves are only formed for sensation, and it is very different with the nerves of voluntary motion." 208.

In one of the experiments re-union was effected by granulation.

In punctures and partial divisions of nerves the reparation is accomplished in a similar way, but even on the first infliction of those injuries the functions of the nerve are little impaired. Though such is the consequence of punctures of nerves in the lower animals, the results are frequently serious in man. We may mention a case which we happened to witness, and one of which we have heard. The first is an instance of puncture of the internal plantar nerve. The patient, a boy, was hastily climbing some iron railings, when the spike of one penetrated his shoe, and wounded the inside of the sole of the foot. In a week or ten days tetanic symptoms supervened, and rapidly proved fatal. On dissection, the spike was found to have punctured the internal plantar nerve, and a nodule of lymph was developed in it or around it. The second case was one of puncture of the peroneal nerve by a pitchfork. The patient, like the former, died of tetanus. Mr. Brodie was in the habit of alluding to this case in his surgical lectures.

"When a portion of a nerve has been removed, the restorative process is set up in the same way as when there has been merely a division of the nerve; but the extremities of the divided portions afterwards present such appearances, as to lead to a supposition that the nerve will never again be restored of the same size it was before. From the repeated experiments I have made, I had been led almost to conclude that a certain portion of a nerve is never restored after its removal, so as to be able to perform its functions; but that this is not always the case, the following circumstance will prove.

A horse had been lame for two years, at the end of which time an inch of each nerve going to the foot was cut out; after this he went very well for six months, when he again became lame, and continued so five months; at the end of this time he appeared to suffer such dreadful pain that he was killed. At the time he was operated on, it was supposed that the disease was the same in both fore-legs, so that portions of the nerves of both of them were removed.

On examining the legs after he was killed, one was very much swelled, especially at the foot, where matter was discharged by several sinuses leading to the coffin bone, which was quite carious.

On further examination, the nerves of this leg were found to have reunited, and the new-formed substance was very large, and appeared to have the same structure as that which forms the bond of union when a nerve has been simply divided. The nerves, above the place where they were divided, were found to be much larger than those of the opposite leg in the same place. In the opposite leg, in which there did not appear to be much disease, the nerves had reunited, but the bond of union was not so large as in the other leg." 210.

Mr. Swan is of opinion that the functions of the nerves were again performed through the medium of the new substance. As this is not usually the case when so much as an inch of the nerve has been removed, he concludes that the restoration was due to the irritation occasioned by the disease in the foot. This is certainly a plausible conjecture, but the foot in which there was not "much disease," is said to have also displayed reunion, though not to so great an extent as the other. From some of the experiments it would seem, that disease of the parts *below* the nerve divided or injured is not a very unfrequent occurrence. This may contribute to increase the difficulty of admitting Mr. Swan's ingenious explanation.

The following observations of our author may be added.

"It appears to me that a reproduction of a portion of a nerve is not accomplished without the greatest difficulty, except where there are very frequent communications with other nerves, or except a much increased action of the blood vessels exists in consequence of a diseased state of the part in which the nerve is situated, as in the case related by Mr. Abernethy, where a portion of one of the digital nerves was removed. When a portion of a nerve has been removed, if its reproduction were a desirable object, this circumstance of its growth in diseased limbs makes it probable that it might be much assisted by irritating frictions, electricity, &c.

Although a large portion of a nerve is seldom restored, yet in some instances new nerves are formed to keep up a communication with the brain. In the eighteenth experiment I observed this for the first time. These new nerves had not that almost transparent appearance that the bond of union has, but were white, and exactly like nerves. It appears extraordinary that entirely new nerves should be formed, but it is not more so than that new arteries should be produced, as Dr. Parry has, I think, satisfactorily demonstrated." 212.

The only experiments to which we shall make individual reference are some which Mr. Swan performed upon the nervus vagus in the neck. They were six in number. In three the nerve was tied—in two divided—and in two a portion was excised. We shall offer an abstract of the consequences which were noticed.

In the first experiment each nervus vagus of a dog was tied with a ligature of silk. The general symptoms were a tendency to vomiting—indisposition to eat and a readiness to drink—agitation—and some dyspnœa. On the fifth day the dog died.

The nerve on the right side was nearly divided by the ligature, the neurilema was thickened and quite ulcerated through ; also the greatest part of the medullary portion, or that forming the central part of the nerve was ulcerated through, so that the two ends just hung together. The two portions of this nerve were more vascular than usual to some distance, but this diminished gradually in both portions, with the distance from the ligature. The portion of the nerve on the left side included in the thread was more inclosed in coagulable lymph than that of the right side, and this lymph appeared vascular, so that the nerve seemed enlarged at this part, and the ligature had not pressed it so completely as to entirely cut off the communication between the two ends. The vascularity of the divided extremities, and in the course of the nerve, was the same as in the other side. The inferior cervical ganglia of the sympathetic, and the first dorsal, especially on the left side, with the semilunar ganglia, displayed very slight vascularity.

The pleura was inflamed, and smeared with purulent matter. The right lung was hepatized, but the left was so in a very slight degree. The inner portion of the pericardium was smeared with a thick glutinous matter, nearly the same as the pleura. The mucous membrane of the larynx and trachea was vascular in a slight degree, and presented some muco-purulent matter with a substance resembling coagulable lymph.

The stomach was much contracted, and a dark vascular spot, of the size of a shilling was observed on its mucous coat at its cardiac end. It contained, as did the intestines, a yellow mucus, principally bile.

In experiment 2, a ligature consisting of two threads was twice passed at the same spot through the right nervus vagus of a large dog. In 21 days the dog was dull, weak, and thinner. On the 28th day the left eye was turned slightly upwards and outwards and was rather watery. In ten days more he was killed by prussic acid.

On examination, the ligature had been thrown off, the nerve was thickened, and very firmly and rather extensively connected with the surrounding parts. The viscera of the chest were natural. The left thoracic ganglia of the sympathetic nerve were not so white as the right. The inner coat of the stomach was reddened as well as that of the intestines, but there were not any ulcers.

In another experiment no symptoms ensued, and the dog made his escape.

In the fourth experiment the right nervous vagus of a fowl was divided. It was killed three days afterwards. The examination presents no feature of interest.

In the fifth experiment each nervus vagus of a fowl was divided. Great dyspnœa immediately succeeded and continued during the day, but was diminished on the following. It ate some oats and was much purged. On the sixth day it was found dead. For the first three days it was fed on oats; for the last on barley, boiled potatoes, and bran.

On examination, the extremities of both nerves were very near each other, enlarged into little bulbs, and surrounded by adhesive matter. The lungs were expanded on both sides, and somewhat redder than in the preceding experiment. The lower part of the œsophagus contained a little food, and the crop was very full of barley, in a swoln state, and some potato. The passage to the gizzard contained the same food mixed with much bile, and some of this fluid was also in the lower part of the crop. In the gizzard there was a small quantity of semi-fluid matter, with much bile, which had a dark green colour. There were fluid fæces in the upper part of the intestines, but only mucus in the lower, except the cloaca. The gall bladder was greatly distended with dark-coloured bile. It may be remarked that no oats were found in the alimentary canal, but barley only.

The fact last mentioned is not undeserving of attention. The food which had been taken on the three days immediately succeeding the division of the nerves was found to be digested, a circumstance tending to prove that the influence of the nervi vagi on the function of digestion is not so important as some persons have imagined.

In the sixth experiment five-eighths of an inch of the left nervus vagus were removed from a large terrier. The principal symptoms with which he was long troubled were sickness, an indistinctness of barking, and loss of flesh; these gradually and materially went off. A year after the operation he barked loudly, but not perfectly; he frequently vomited up his food and ate it again, but he was well fed, so that his body was sufficiently fleshy; and he breathed well.

At the termination of a year Mr. Swan divided the right nervus vagus. The symptoms that followed were vomiting, agitation, and some difficulty of breathing. On the third day he died.

The right side of the chest was much more capacious than the left. The right lung was not collapsed, was purple in patches, and more solid than natural; the left lung was more solidified, and less in size than the right, and displayed similar purple patches.

The left nervus vagus, from which a part had been removed was united by apparently new small nerves, and a twig went from the branch of the first cervical, which joins the descending branch of the ninth to the inferior portion. The right was united by a coagulum of blood. The left recurrent nerve was much less than the right.

The stomach was excessively contracted. The viscera were healthy with the exception of a few red spots on the mucous membrane of the superior part of the intestines, which presented the appearance of ecchymosis.

The blood was quite black in the aorta.

In the seventh experiment Mr. Swan cut out a portion measuring a quarter of an inch from the right nervus vagus of a white rabbit. It was hardly affected by the operation, and had litters of young ones afterwards.

In a year and eight months after the former operation Mr. Swan cut out a portion from the left nervus vagus, and removed every nervous twig near the carotid.

The chief subsequent symptom was dyspnœa. It was found dead on the eighth day.

The right side of the chest was flat, and the left side was considerably larger than the right. Both lungs were diseased, the upper two-thirds of each contained many tubercles, a few of which were in a state of suppuration; the lower portion of each lung was free from tubercles. There were purple patches on the sound part of each lung. There were many hydatids in the abdomen, but all the other viscera were sound.

The right trunk of the par vagum had united, and although the bond of union was smaller than the original part, it had the appearance of nerve.

In this and the preceding experiment an interval of twelve months or upwards had elapsed between the two operations on the nerves. In the one instance new nervous twigs appear to have been formed to supply the place of the portion excised—in the other a nervous cord of diminished diameter seems to have been made the substitute. Yet in both experiments the operation on the remaining nerve (division in the one instance, excision of a part in the other) was followed by the death of the animal. The author thinks that in the last experiment the result would have been more fortunate had tubercles not existed in the lungs. This is of course incapable of demonstration.

Ligature of both nerves was followed by death and inflammation of the lungs and pleura.

The passage of a ligature through one nerve does not seem to have occasioned any very prominent or fatal symptoms.

The division of a single nerve was scarcely productive of appreciable effects.

The division of both nerves occasioned death, preceded by symptoms of dyspnœa. On dissection the lungs were found expanded and reddened. Bile had been freely secreted, and food had been digested.

It is needless and would be useless to draw further inferences from these few facts. The lungs would appear to be more vitally affected than the stomach by experiments on the nervus vagus.

Experiments on this nerve may naturally lead us to reflect on its diseases, if such are appreciable by symptoms or dissection. Mr. Swan has devoted a chapter (the tenth) to their elucidation. It cannot be expected that much new light should be shed on such a subject, and we feel no surprise at the failure of Mr. Swan to convey any satisfactory or positive information. The prominent fact is one which, though diffuse, we will transcribe without material abridgement, as we do not coincide with the opinions of the author on its nature.

The Rev. Mr. Deacon, aged *sixty-two*, had suffered from gout from the early age of seventeen years. For this he had taken considerable quantities of the eau medicinale and Dr. Wilson's medicine. The functions of the stomach had been much impaired for the last seven years.

"Nov. 19, he began to have a difficulty of breathing, which was only perfectly relieved either by a fit of the gout, or the vinous infusion of the colchicum root. When the difficulty of breathing was the worst, he made a whistling noise, as though the glottis was contracted. For some time he was quite free from it. But in the Summer of 1821 he had a violent attack of it, which was thought to have been produced by cold, when the gout came on and relieved him; he took the vinous infusion of colchicum, which removed the gout, but it purged him so violently, that an opiate was given him to restrain it. He began to sleep from this time, and continued so three weeks, except he was roused, when he seemed to know every one about him, but had neither recollection nor judgment beyond this. Leeches, blisters, &c. seemed to relieve him in some degree, but he was not materially better until he took the vinous infusion of colchicum again; after which he continued to mend gradually, but the difficulty of breathing soon returned; and though it yielded once or twice more to the vinous infusion of the colchicum, which always purged him, no lasting good effect was produced. The difficulty of breathing continued, with different degrees of violence, till the time of his death. He never had pain in the chest. His stomach remained in the same craving and insensible state to the last. His body for months had been becoming more and more emaciated. His pulse was generally natural, but very strong. For several weeks he could not take opiates of any sort, or in any quantity, as they made him so uncomfortable. About ten days before he died, he had a teacupful of blood taken from the arm, which somewhat relieved his breathing. He, at this time, had much gouty inflammation in his hands. The blood was very much cupped, and had a strong buffy coat, but the next day he was so very faint, that he could with difficulty sit in his chair. He had a cough, which was occasionally troublesome. As nothing seemed to relieve him, and as his state appeared to me to be much approaching that of an animal whose eighth pair of nerves had been divided, he was galvanised. He thought the two first trials gave him some relief. A few nights before he died he was taken with difficulty of breathing to such a degree, and seemed so exhausted, that some wine was given him, and galvanism was again tried. After the first ten minutes, the noise in his breathing left him, and he kept breathing more and more easily, so that when the galvanism had been used for half an hour, he laid down and slept better for several hours, than he had done for some time before. The galvanism was repeated the next day, and he thought himself relieved by it; but this relief was of short duration, for his breathing soon became as bad as ever, and he died a few days after, on the 22d of September.

Within the last three weeks he had been obliged to rise in the night, and sit up a great part of it. Owing to this his legs swelled a little, as he was unable to have them in a horizontal position when he sat up, in consequence of his knees being much contracted. About ten days before he died, purple spots appeared on his feet, and then on the rest of his body; these, however, disappeared entirely in three or four days.

His symptoms never were those strongly marking hydrothorax, and his countenance had not the expression usual in this disease." 173.

We suspect that the majority of those accustomed to morbid anatomy and the practice of medicine, would recognize the symptoms and anticipate the existence of disease of the heart and effusion in the chest, in the preceding instance. Their grosser apprehension would fail to be struck by the lighter shadows that arrested the attention and excited the reflection of the gentleman before us. They would see in the advanced age of the patient, in the paroxysms of dyspnœa, the orthopnœa, the obvious tendency to anasarca, and the disposition to petechiæ, the signs and the concomitants of what is improperly termed hydrothorax.* They would look with confidence to the examination of the body, and to this we will now refer.

"On opening the abdomen every thing appeared sound. The outside of the stomach was covered with an unusual quantity of veins, but the inside of this viscus had nothing particular in its appearance.

On opening the chest there was much fat. The heart was enlarged and fat, but otherwise every thing about it had a healthy appearance. In the pulmonary artery one sesamoid body was larger than the rest; and in the aorta, one of these bodies was not situated at the edge of the valve, but about its middle. Each side of the chest contained about two pints of a dark-coloured fluid. The lungs were not collapsed, but appeared otherwise healthy.

On tracing the par vagum from the middle of the neck each nerve was flabby, and much smaller than natural, and felt like nerves removed from a putrid body after having been soaked in water. The branches distributed to the lungs appeared as usual, as well as the continuations of the nerves, nearly as far as the termination of the œsophagus, when they were found redder and thicker than usual, and had not a healthy appearance. The left nerve was smaller than the right.

A similar state of the lungs has been generally observed after a division of the par vagum in rabbits." 175.

Examination may be said to have disclosed enlargement with a fatty condition of the heart—a quart of dark fluid in each side of the chest—and a flabbiness with apparent diminution of size in the par vagum.

The matter of fact observers to whom we have alluded would probably appeal with confidence and triumph to these results. They would argue with some degree of plausibility that the state of the heart and the fluid in the chest corresponded with the nature of the symptoms during life—that such accordances are daily witnessed—that flabbiness and diminution of size of the par vagum are lesions of very questionable character, as we cannot pronounce what should be the bulk, nor what is the consistence of this or of other nerves in different subjects and in different diseases.

Mr. Swan would appear to be prepared for such objections. He subjoins the following statements:

"In order to be better satisfied with regard to the diminution of the size of the par vagum in the preceding case, I was led to compare it with that in other subjects; and in dissecting two destroyed by consumption, the left lungs were diseased in a much greater degree than the right; both trunks of the par vagum were smaller than usual, and especially when compared with those of a subject

---

* We need scarcely explain this allusion. Hydrothorax is commonly a symptom and a consequence of organic alterations of the heart. The symptoms said to be those of hydrothorax may exist when it is absent, and result from the condition of the heart. Some of the symptoms, for example, orthopnœa, are undoubtedly aggravated on the supervention of effusion.

destroyed by empyema in which the lungs were sound; and the left trunk was smaller than the right. In both of these subjects, as well as in another, very considerable disease existed in the intestines. In one I examined the alimentary canal from one end to the other, and through its whole length beyond the stomach, very little space was left where the ulcerations of the mucous membrane did not exist, most of them varying in size from a pea to half-a-crown." 175.

Mr. Swan's analogy would seem to tell against himself. The diminution of the nervi vagi in the case of phthisis pulmonalis must be viewed, if regarded with interest at all, as a consequence or a cause of that disease. If deemed to be the former, it might also be the consequence of thoracic disease in the case at issue—if the latter, we presume that the cautious reader would pause for proof and demand investigation. Mr. Swan would hesitate at giving utterance to so startling an hypothesis.

In examining the bodies of those who have died of consumption, Mr. Swan has frequently found the par vagum, and particularly the posterior pulmonary plexuses incroached on by enlarged absorbent glands. He believes that these glands have often been affected before the lungs have become diseased, and by their progressive enlargements have implicated the nerves and excited irritation in the lungs.

"In consumption, I believe, many of the distressing symptoms are frequently produced by the stretching of these nerves by enlarged glands. In these cases there have not been any decidedly inflammatory symptoms or much quickness of the pulse. There has been a troublesome cough without much expectoration. The appetite has been whimsical. There have been frequent attacks of difficulty of breathing, sometimes of oppression with a sense of suffocation, and these symptoms have been relieved by some stimulus, as a small quantity of brandy and water, or ammoniated tincture of valerian, and brought on again by any thing that disagreed with the stomach. There has been a distressing nausea, which was relieved by opiates. In one of these cases, a gland as big as a pigeon's egg was found stretching the left trunk of the par vagum and the branches forming the posterior pulmonary plexus." 177.

Lobstein describes the same stretching of the par vagum by absorbent glands. He has often found stony concretions adhere to the nerves, and be separable only by force. He has found the same thing in the solar plexus, where it approaches the supra renal gland, and in the trunk of the par vagum itself separating its fibrils, but not impairing the functions of the stomach and the lungs.

"I examined a tumour extending towards the epigastric region and firmly adhering to the small curvature of the stomach. Both trunks of the par vagum were broken, the right one formed a lengthened ganglion from which three tender branches went off. The patient, a man forty years old, was seized with sudden heartburn, and tormented with excruciating pains in the back and the interscapular region. To these symptoms were added an obstinate constipation and the most severe tormina, from which he died after a space of seven weeks. This disease was certainly of long standing and not marked by any symptoms, but from the time the par vagum was daily stretched and then torn through by the tumour, the most violent symptoms were manifested. It could not have happened otherwise, than that the twitchings, conveyed to the semilunar ganglion and the right trunk of the sympathetic nerve, should have produced the dreadful pain in the abdomen and back." 178.

Mr. Swan makes allusion to the ulceration of the intestines that occurs in the latter stages of consumption. He accounts for this by supposing that their mucous membrane effuses a poisonous exhalation similar to that which is generated in the lungs.

We cannot deem this a felicitous idea. We see no more reason for supposing that a poisonous exhalation occurs in phthisis than in fever, idiopathic or hectic, where ulcerations of the bowels are frequently, nay constantly, observed. The morbid anatomist is sufficiently aware that a febrile state of system cannot long exist, no matter what its cause may have been, without a marked disposition to inflammation and ulceration of the follicles of the intestines. Why this should be we will not now inquire, but the circumstances under which it is observed are so numerous and so varied, that the doctrine of a poisonous exhalation could only be admitted or applied with the greatest difficulty and the greatest danger.

Our space is now so nearly exhausted that necessity compels us to select the shortest chapter to complete this notice. It treats of tumours in nerves.

A tumour in a nerve causes very violent pain. It is generally solid and formed by the interposition of adhesive matter between the fibrils; but sometimes it is a cyst containing a gelatinous matter, which has been generated in the substance of the nerve. It varies in size, from a grain of wheat to a substance of considerable magnitude. It may be distinguished from all other tumours by the excessive pain produced by pressure, and by the extension of this in the course of the affected nerve. It is generally moveable from side to side only as the upper and lower extremities are more or less confined by the nerve. If it be not removed the violent pain gradually wears away the strength of the sufferer, and he dies at last completely exhausted.

Epilepsy is not uncommonly produced by a tumour in a nerve, and removed by its removal.

Small tumours are not unfrequently seated in the cutaneous nerves, and are felt immediately beneath the skin. Most surgeons in extensive or in hospital practice must have met with cases of this description. Mr. Swan relates one or two.

*Case.* Mrs. H. had felt for two years a pain in a small spot about the middle of the leg. A small tumour could be then distinguished, which, when pressed, was extremely painful. In seven years it had attained the size of a large pea. The suffering was very great, and was always brought on and aggravated by surprise, fear, or any affection of the mind, and likewise by cold. Keeping the whole body warm always relieved the pain. A surgeon divided, the skin over it, and kept the wound open for three months by caustic frequently applied. This treatment was not productive of benefit. Mr. Swan cut out the tumour with the surrounding portion of skin. When divided it presented a cartilaginous appearance, and a cutaneous nerve was seen passing between it and the skin, and an expansion of the nerve was spread over it. After its removal all the distressing symptoms left her, and never returned.

M. Beclard, when a student, had a small and very painful tumour in the leg, about the size of a grain of wheat, which disappeared spontaneously some months after he had removed from a very unhealthy apartment he had for a long time previously occupied.

"Small tumours or enlargements of various nerves are observed on dissection, but as the history of the subjects is not known, it can only be conjectured that these might have occasioned pains that could not be satisfactorily accounted for during the patient's life. In one subject there was a considerable enlargement of the digital nerve supplying the inner edge of each great toe; and at the outer side of each foot, at the junction of the metatarsal bone with the first phalanx, there was a ganglion or bursa the size of a horse-bean, which adhered to a digital nerve, and from this an enlarged branch was continued into its cavity. These diseases are the cause of very great inconvenience, and ought not to be roughly treated." 89.

We might mention some cases of small tumours, of the size of peas, or smaller, in cutaneous nerves. They produce extreme and sometimes agonizing pain, generally experienced in the course of the nervous branch.

Mr. Swan observes that the only effectual remedy is excision of the tumour and of some of the nerve above it and below it. This removal of part of a cutaneous nerve is of little moment.

But tumours or other morbid conditions implicate nerves of more magnitude and consequence. A case of Sir Charles Bell's, of Dr. Denmark's, and of Sir Everard Home's, is severally quoted by our author.

*Case.* A man is stated by the first of these gentlemen to have bruised the back, and to have apparently recovered from the injury. Some time afterwards he began to be much troubled with a violent pain in his foot, which he suffered for two years. At the end of this time a tumour was discovered in the ham, which, when pressed on, did not give any particular pain, but rather, a sense of pricking numbness down the leg. He was then in a dying state, and only lived a few days afterwards.

On dissection, some nerves were found running over the tumour. The sciatic nerve entered into the substance of the tumour, but the fibular nerve, though close on it, was not incorporated with it.

The case of Dr. Denmark is probably familiar to the majority of our readers. Yet we will introduce it on account of its intrinsic interest.

*Case.* A man had a small tumour in the arm, which followed a gun-shot wound, produced very violent symptoms, and was cured by amputation of the arm.

On dissection of the limb the median nerve seemed to be blended with, and intimately attached to, the wounded parts for the space of an inch. It had been wounded, and at the place of the injury was thickened to twice its natural diameter, and seemed as if contracted in length. On dividing the fibres in the posterior part of the wounded nerve, a small portion of nerve was found firmly imbedded in it. The nerve was evidently thickened both above and below the wound.

Mr. Swan now enters on an important question. Is it better to excise a portion of the nerve affected with a tumour or disease—to attempt the removal of the latter alone—or simply to divide the nerve above the tumour.

The latter proposal may be speedily disposed of. It is only a fruitless and a temporary expedient. As soon as the divided portions reunite, the conditions of the nerve and of the malady are nearly re-established, as before.

The effects of excision of the tumour are in some degree exemplified by a case which occurred to Sir Everard Home. Here we must abruptly pause. The subject will be resumed in our succeeding Number.

